# *Phellinus linteus* suppresses growth, angiogenesis and invasive behaviour of breast cancer cells through the inhibition of AKT signalling

**DOI:** 10.1038/sj.bjc.6604319

**Published:** 2008-03-25

**Authors:** D Sliva, A Jedinak, J Kawasaki, K Harvey, V Slivova

**Affiliations:** 1 Cancer Research Laboratory, Methodist Research Institute, 1800 N Capitol Ave, E504, Indianapolis, IN 46202, USA; 2 Department of Medicine, Indiana University, Indianapolis, IN, USA; 3 Indiana University Simon Cancer Center, School of Medicine, Indiana University, Indianapolis, IN, USA

**Keywords:** *Phellinus linteus*, invasiveness, angiogenesis, AKT

## Abstract

The antitumour activity of a medicinal mushroom *Phellinus linteus* (PL), through the stimulation of immune system or the induction of apoptosis, has been recently described. However, the molecular mechanisms responsible for the inhibition of invasive behaviour of cancer cells remain to be addressed. In the present study, we demonstrate that PL inhibits proliferation (anchorage-dependent growth) as well as colony formation (anchorage-independent growth) of highly invasive human breast cancer cells. The growth inhibition of MDA-MB-231 cells is mediated by the cell cycle arrest at S phase through the upregulation of p27^Kip1^ expression. *Phellinus linteus* also suppressed invasive behaviour of MDA-MB-231 cells by the inhibition of cell adhesion, cell migration and cell invasion through the suppression of secretion of urokinase-plasminogen activator from breast cancer cells. In addition, PL markedly inhibited the early event in angiogenesis, capillary morphogenesis of the human aortic endothelial cells, through the downregulation of secretion of vascular endothelial growth factor from MDA-MB-231 cells. These effects are mediated by the inhibition of serine-threonine kinase AKT signalling, because PL suppressed phosphorylation of AKT at Thr^308^ and Ser^473^ in breast cancer cells. Taken together, our study suggests potential therapeutic effect of PL against invasive breast cancer.

Despite the fact that breast cancer mortality of breast cancer decreased by 22.9% from 1991 to 2003, breast cancer remains the leading cause of cancer death among 20- to 59-year-old US women with estimated 40 460 new death in 2007 (Jemal *et al*, 2007). One of the reasons for such a high mortality is the invasive behaviour of cancer cells, which results in the metastasis of breast cancer. Cancer metastasis consists of several interdependent processes including uncontrolled cell proliferation, invasion through surrounding tissues, migration to the distant sites of the human body, and adhesion, invasion and colonisation of other organs and tissues (Price *et al*, 1997). Tumour growth and metastasis also require angiogenesis, the formation of blood vessels by capillaries sprouting from pre-existing vessels (Folkman, 1995). Therefore, inhibition of growth, invasive behaviour and cancer cell-mediated angiogenesis will lead to the suppression of cancer metastasis and would further increase survival of breast cancer patients.

*Phellinus linteus* (PL) is a basidiomycete fungus, located mainly in tropical America, Africa and Asia, where it gained significant recognition as medicinal mushroom in the traditional Oriental medicine (Dai and Xu, 1998). The biologically active compounds isolated from PL are polysaccharides (Song *et al*, 1995), acidic proteo-heteroglycans with mixed *α*- and *β*-linkages, and a (1 → 6)-branched type (1 → 3)-glycan (Kim *et al*, 2003b). These complex polysaccharides have been detected in a variety of different mushroom species and linked to the immunostimulatory and antitumour activities (Wasser, 2002). Therefore, PL stimulated proliferation of T lymphocytes and activated B cells (Kim *et al*, 1996, 2003c), and induced maturation of bone marrow-derived dendritic cells (Park *et al*, 2003) and the macrophage response (Kim *et al*, 2003a). On the other hand, PL demonstrated anti-inflammatory effects in lipopolysaccharide-stimulated macrophages (Kim *et al*, 2006). The direct anticancer effect of PL has been demonstrated by the inhibition of invasive melanoma B16BL6 cells through the downregulation of mRNA level of urokinase-plasminogen activator (uPA), and by the inhibition of pulmonary metastasis in mice (Lee *et al*, 2005). *Phellinus linteus* suppressed proliferation by the inhibition of cyclin-dependent kinases cdk2, 4 and 6, and induced apoptosis through the activation of caspase 3 in lung cancer cells (Guo *et al*, 2007) and apoptosis of prostate cancer cells (Collins *et al*, 2006; Zhu *et al*, 2007).

In the present study, we evaluated the effect of PL on the highly invasive and metastatic human breast cancer cells. Here, we show that PL inhibits proliferation (anchorage-dependent growth) as well as colony formation (anchorage-independent growth) of highly invasive human breast cancer cells. In addition, PL also suppresses invasive behaviour of MDA-MB-231 cells by the inhibition of cell adhesion, cell migration and cell invasion. Finally, PL suppressed breast cancer cell-mediated angiogenesis of endothelial cells *in vitro*. Collectively, our study suggests the mechanism(s) employed for the inhibition of proliferation, invasive behaviour and angiogenesis of invasive breast cancer cells by an extract from medicinal mushroom PL.

## MATERIALS AND METHODS

### Cell culture and reagents

Human breast cancer cells (MCF-7, MDA-MB-231) and human prostate cancer cells (PC-3, LNCaP) were obtained from ATCC (Manassas, VA, USA). MCF-7 and MDA-MB-231 cells were maintained in DMEM medium, PC-3 cells were maintained in F-12 medium and LNCaP cells were maintained in RPMI 1640 medium. All media contained penicillin (50 U ml^−1^), streptomycin (50 U ml^−1^), and 10% fetal bovine serum (FBS). Media and supplements came from GIBCO BRL (Grand Island, NY, USA). Fetal bovine serum was obtained from Hyclone (Logan, UT, USA). Aqueus extract of PL was supplied by the Maitake Products Inc. (Paramus, NJ, USA). Stock solution was prepared by dissolving PL in sterile water at a concentration 50 mg ml^−1^ and stored at 4°C. AKT inhibitors LY294002 and AKT inhibitor III were obtained from Calbiochem (San Diego, CA, USA).

### Cell proliferation assay

Cell proliferation was determined by the tetrazolium salt method, according to the manufacturer's instructions (Promega, Madison, WI, USA). Briefly, cancer cells were cultured in a 96-well plate and treated at indicated times with PL (0–1.0 mg ml^−1^). At the end of the incubation period, the cells were harvested and absorption was determined with an ELISA plate reader at 570 nm, as described (Jiang *et al*, 2004b). Data points represent mean±s.d. in the representative experiment of triplicate determinations. Similar results were obtained in two independent experiments.

### Cell viability

Cell viability of MCF-7 and MDA-MB-231 cells was determined after incubation with PL (0–1.0 mg ml^−1^) for 24, 48 and 72 h by staining with Trypan Blue as described (Sliva *et al*, 2002a).

### Anchorage-independent growth

MDA-MB-231 cells were harvested and seeded in six-well plates coated with 1% agarose. Anchorage-independent growth was assessed after incubation for 10–14 days with culture media with or without PL (0–1.0 mg ml^−1^), which were replaced every 4 days. Plates were stained with 0.005% crystal violet, and the colonies were counted manually under a microscope and photographed (Slivova *et al*, 2004).

### Cell cycle analysis

MDA-MB-231 cells (0.75 × 10^6^) were seeded and after 24 h treated with PL (0.5 mg ml^−1^) for the indicated period of time (0–48 h). After incubation, the cells were harvested by trypsinisation, washed with Dulbecco's phosphate-buffered saline containing 2% FBS, and resuspended in propidium iodine (50 *μ*g ml^−1^). Cell cycle analysis was performed on a FACStar^PLUS^ flow cytometer (Becton-Dickinson, San Jose, CA, USA), as previously described (Sliva *et al*, 2001). Data are the mean±s.d. from three independent experiments.

### Cell adhesion, migration and invasion assays

Cell adhesion was performed with Cytomatrix Adhesion Strips coated with human vitronectin (Chemicon International, Temecula, CA, USA). Briefly, MDA-MB-231 cells were treated with PL (0–0.5 mg ml^−1^) for 24 h, harvested and counted. Cell adhesion was determined after 1.5 h of incubation at 37°C (Lloyd *et al*, 2003). Cell migration of MDA-MB-231 cells treated with PL (0–1.0 mg ml^−1^) was assessed in Transwell chambers in the DMEM medium containing 10% FBS (Slivova *et al*, 2004). Invasion of MDA-MB-231 cells treated with PL (0–1.0 mg ml^−1^) was assessed in Transwell chambers coated with 100 *μ*l of Matrigel™ (BD Biosciences, Bedford, MA, USA) diluted 1:4 with DMEM, after 72 h of incubation (Slivova *et al*, 2004).

### *In vitro* endothelial cell morphogenesis assay (capillary morphogenesis)

Human aortic endothelial cell (HAEC) differentiation into ‘capillary-like’ structures was observed using a two-dimensional Matrigel-based assay as we described previously (Harvey *et al*, 2002). Initially, 200 *μ*l of ice-cold growth factor-reduced Matrigel (Becton Dickinson Labware, Bedford, MA, USA), an extracellular matrix preparation derived from the Engelbreth–Holm–Swarm tumour, was placed into each well of a 24-well tissue culture-treated plate. Human aortic endothelial cells were harvested, resuspended in serum-free EBM media and plated at 3.5 × 10^4^ cells per well-coated with Matrigel. Human aortic endothelial cells were further incubated with PL (0–0.5 mg ml^−1^) or with conditioned media from MDA-MB-231 cells, which were prepared by the incubation of MDA-MB-231 cells in the presence of PL (0–0.5 mg ml^−1^) for 24 h. Endothelial HAE cells differentiated into capillary-like structures within 16 h of incubation at 37°C in the presence of 5% CO_2_. These structures were examined microscopically ( × 40) using an inverted Olympus CK40 microscope. To facilitate analysis of the structures, non-adherent cells incorporated in excess medium were removed from each well prior to quantitative analysis. Photomicrographs were taken to assess the extent of capillary-like structural formation. Quantification of the capillary-like structures was performed counting the number of nodes per field, where a node is defined as an intersection of at least three cells. Each sample was assayed in triplicate and reproduced in at least two additional experiments.

### Western blot analysis

MDA-MB-231 cells were treated with PL (0–1.0 mg ml^−1^) for 24–72 h as indicated in the text and whole-cell extracts prepared as described previously (Sliva *et al*, 2002a, 2002b). Equal amounts of proteins (20 *μ*g per lane) were separated on NuPAGE 4–12% Bis-Tris gel (Invitrogen, Carlsbad, CA, USA) and transferred to a PVDF membrane (Millipore, Bedford, MA, USA). The protein expression was detected with the corresponding primary antibodies: anti-cyclin-D1, anti-cyclin-E, anti-cyclin-A, anti-cdk2, anti-cdk4, anti-p21, anti-p27, anti-*β*-actin, (Santa, Cruz Biotechnology, Santa Cruz, CA, USA), anti-AKT, anti-phospho-AKT (Thr^308^) and anti-phospho-AKT (Ser^473^) (Cell Signaling, Beverly, MA, USA), respectively. Protein expression was visualised using the ECL Western Blotting Detection System (Amersham Biosciences, Buckinghamshire, UK).

### Urokinase-plasminogen activator secretion

DMEM media from MDA-MB-231 cells treated with PL (0–1.0 mg ml^−1^) for 24 h were collected and concentrated, and the secretion of uPA was detected by western blot analysis with anti-uPA antibody (Oncogene Research Products, Cambridge, MA, USA), as described (Sliva *et al*, 2002b).

### Densitometric analysis

Autoradiograms of the western blots were scanned with HP scanjet 5470c scanner. The optical densities of p27, phospho-AKT (Thr^308^), phospho-AKT (Ser^473^), AKT, *β*-actin and uPA proteins on the films were quantified and analysed with the UN-SCAN-IT software (Silk Scientific, Orem, UT, USA). The ratios of p27/*β*-actin and phosphoAkt/Akt were calculated by standardising the ratios of each control to the unit value.

### Vascular endothelial growth factor secretion

MDA-MB-231 cells were treated with PL (0–0.5 mg ml^−1^) for 24 h, cell media collected and secretion of vascular endothelial growth factor (VEGF) was determined using a respective Quantikine ELISA kit (R&D Systems, Minneapolis, MN, USA) according to the manufacturer's instructions.

### Statistical analysis

Data are presented as means±s.d. Statistical comparison between the control group (0 *μ*g ml^−1^ of PL) and groups with different PL doses was carried out using two-sided Student's *t*-tests. The value of *P*<0.05 was considered to be significant.

## RESULTS

### *Phellinus linteus* suppresses proliferation and colony formation of highly invasive breast cancer cells

**Figure 1:**
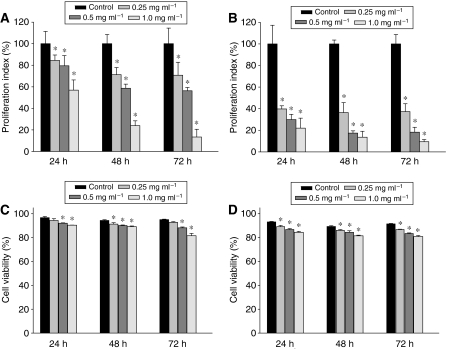
Effect of PL on proliferation of breast cancer cells. Proliferation: (**A**) MDA-MB-231, (**B**) MCF-7 cells were treated with PL (0–1.0 mg ml^−1^) for 24, 48 and 72 h. Cell proliferation was determined as described in Materials and Methods. Data are the means±s.d. of triplicate determinations. Similar results were obtained in at least two additional experiments. ^*^P<0.05. Viability: (**C**) MDA-MB-231, (**D**) MCF-7 cells were treated with PL (0–1.0 mg ml^−1^) for 24, 48 and 72 h. Cell viability was determined by Trypan blue staining as described in Materials and Methods. Data are the means±s.d. of triplicate determinations. Similar results were obtained in at least one additional experiment. ^*^P<0.05.

Invasive behaviour of cancer cells is directly linked to their metastatic potential resulting in the high cancer mortality. Therefore, we evaluated if PL inhibits growth of highly invasive (MDA-MB-231) and poorly invasive (MCF-7) breast cancer cells. As seen in Figure 1, increased concentration of PL (0–1.0 mg ml^−1^) markedly suppressed proliferation of MDA-MB-231 as well as MCF-7 cells in a dose- and time-dependent manner. Nevertheless, the effect of PL on poorly invasive cells was more pronounced, because the concentration 0.25, 0.5 and 1.0 mg ml^−1^ of PL suppressed proliferation of MCF-7 cells by 60.2, 70.1 and 78.0%, respectively (Figure 1B), whereas the same concentration suppressed proliferation of MDA-MB-231 cells by 15.5, 21.5 and 43.1%, respectively (Figure 1A), after 24 h of incubation. The same sensitivity of MCF-7 cells was evident also after additional 48 and 72 h of incubation, where only the highest concentration of PL (1.0 mg ml^−1^) suppressed proliferation of poorly invasive and highly invasive breast cancer cells with the same potency (Figure 1). To determine if the effect of PL on cancer cells is cytotoxic or cytostatic, we evaluated the cell viability after 24, 48 and 72 h of PL treatment. Although PL decreased the viability of MDA-MB-231 and MCF-7 cells, the strongest inhibition of cell viability at the highest used concentration of PL (1.0 mg ml^−1^) after 72 h was only 13.5% for MDA-MB-231 cells (Figure 1C) and 10.6% for MCF-7 cells (Figure 1D), whereas the same concentration suppressed proliferation of MDA-MB-231 cells by 86.6% (Figure 1A) and MCF-7 cells by 90.6% (Figure 1B). Therefore, these data suggest that the PL inhibits growth of breast cancer cells predominantly through its cytostatic effect. Interestingly, PL also suppressed proliferation of poorly invasive prostate (LNCaP) and highly invasive prostate (PC-3) cancer cells in a dose- and time-dependent manner, and LNCaP cells were more sensitive to the PL treatment (not shown).

**Figure 2:**
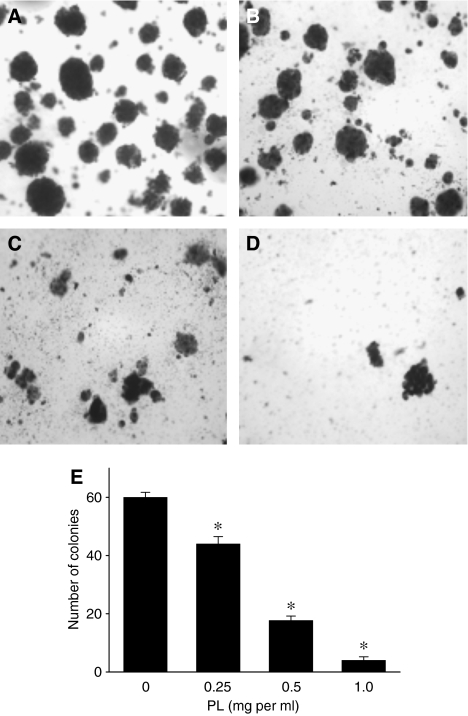
Effect of PL on colony formation of MDA-MB-231 cells. Anchorage-independent growth (colony formation) of MDA-MB-231 cells was assessed on 1% agarose after incubation for 14 days with culture media containing: (**A**) 0 mg ml^−1^ PL, (**B**) 0.25 mg ml^−1^ PL, (**C**) 0.5 mg ml^−1^ PL, (**D**) 1.0 mg ml^−1^ as described in Materials and Methods. (**E**) The number of colonies was determined as described in Materials and Methods. The data are the means±s.d. from three experiments. ^*^P<0.05.

In addition to cell proliferation (anchorage-dependent growth), colony formation (anchorage-independent growth) is one of the typical characteristic of the metastatic potential of cancer cells *in vitro* and strongly correlates with tumorigenesis *in vivo* (Freedman and Shin, 1974). To determine whether PL suppresses colony formation of highly invasive breast cancer cells, we evaluated the anchorage-independent growth of MDA-MB-231 cells. As seen in Figure 2, MDA-MB-231 cells formed colonies on agar after 14 days of incubation, and the presence of increased concentration of PL (0–1.0 mg ml^−1^) resulted in the significant suppression of number of colonies (Figure 2E). Therefore, PL inhibits anchorage-dependent as well as anchorage-independent growth of highly aggressive breast cancer cells.

### *Phellinus linteus* induces cell cycle arrest at S phase

**Figure 3:**
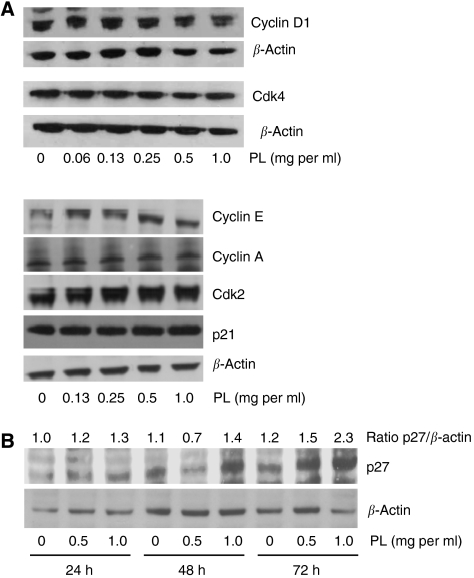
Effect of PL on the expression of cell cycle regulatory proteins. MDA-MB-231 cells were treated with PL (0–1.0 mg ml^−1^) for (**A**) 24 h, or (**B**) 24–48 h and whole-cell extracts were subjected to Western blot analysis. The expression of cyclin D1, cyclin E, cyclin A, cdk2, cdk4, p21 and p27 was evaluated by western blot analysis with their respective antibodies. The equal protein loading was verified with anti-*β*-actin antibody. The results are representative of three separate experiments. The expression level of p27 (ratio p27/*β*-actin) was quantified by densitometry as described in Materials and Methods.

To determine whether the inhibition of cell proliferation is associated with cell cycle arrest, MDA-MB-231 cells were treated for 24 and 48 h with PL (0.5 mg ml^−1^) and analysed by flow cytometry. Cell cycle analysis demonstrated that PL causes cell cycle arrest at S phase of cell cycle (Table 1), where the amount of cells in S phase significantly increased from 27% (control – 0 h) to 34% (24 h) and 44% (48 h). In addition, PL treatment did not induce the amount of cells in sub-G0/G1 phase, further suggesting the cytostatic effect of PL on MDA-MB-231 cells (Table 1). To examine the mechanism responsible for the cell cycle arrest at S phase, we evaluated the expression of cell cycle regulatory proteins (cyclins) and cdks involved in the progression from G1 phase to S phase (Sherr and Roberts, 1999; Ekholm and Reed, 2000). Therefore, MDA-MB-231 cells were treated with PL (0–1.0 mg ml^−1^) for 24 h and the expression of cyclin D1, E, A, cdk4, cdk2, p21 and p27 in cell extracts was evaluated by western blot analysis with respective antibody. Neither of the G1 regulatory proteins cyclin-D1, cdk4 or p21, G1-late phase or G1/S-phase regulatory proteins cyclin E, A and cdk2 demonstrated changes in their expression after the treatment with PL (Figure 3A). Nevertheless, PL (0.5 and 1.0 mg ml^−1^) markedly induced expression of a cyclin-dependent kinase inhibitor p27 (Figure 3B). Therefore, PL inhibits proliferation of breast cancer cells by the upregulation of p27 resulting in S-phase cell cycle arrest.

### *Phellinus linteus* suppresses invasive behaviour of breast cancer cells

**Figure 4:**
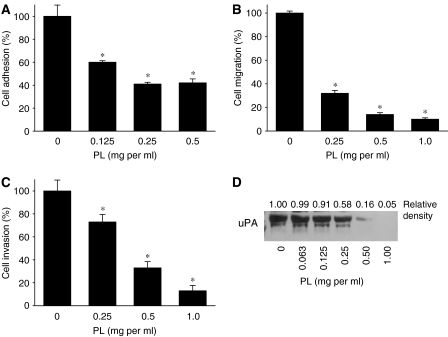
Effect of PL on invasive behaviour of MDA-MB-231 cells. (**A**) Cell adhesion. MDA-MB-231 cells were treated with PL (0–0.5 mg ml^−1^) for 24 h and cell adhesion to vitronectin determined as described in Materials and Methods. Each bar represents the mean±s.d. of three experiments. ^*^P<0.05. (**B**) Cell migration. Cell was determined after 5 h of incubation in the presence of PL (0–1.0 mg ml^−1^) in Boyden Chambers as described in Materials and Methods. Each bar represents the mean±s.d. of three experiments. ^*^P<0.05. (**C**) Cell invasion. Cell invasion was determined after 24 h of incubation in the presence of PL (0–1.0 mg ml^−1^) in Boyden Chambers coated with Matrigel as described in Materials and Methods. Each bar represents the mean±s.d. of three experiments. ^*^P<0.05. (**D**) uPA secretion. MDA-MB-231 cells were treated with PL (0–1.0 mg ml^−1^) for 24 h, and the expression of uPA detected in conditioned media with anti-uPA antibody by western blot analysis as described in Materials and Methods. The secretion of uPA was quantified by densitometry as described in Materials and Methods. The results are representative of three separate experiments.

The ability of cancers to metastasise is directly associated to cell adhesion, migration and invasion. Integrin receptor *α*_V_*β*_3_ is involved in adhesion of breast cancer cells through its interaction with extracellular matrix (ECM) protein vitronectin (Wong *et al*, 1998). To investigate if PL affects adhesion of invasive breast cancer cells, MDA-MB-231 cells were pretreated with PL (0–0.5 mg ml^−1^) for 24 h and their adhesion to vitronectin was determined. As seen in Figure 4A, adhesion of MDA-MB-231 cells was markedly suppressed by the PL treatment. Next, we evaluated if PL also inhibits cell migration. MDA-MB-231 cells were pretreated with PL (0–1.0 mg ml^−1^) for 1 h and cell migration was determined after additional 5 h of incubation. As seen in Figure 4B, PL also markedly suppressed migration of breast cancer cells in a dose-dependent manner. Finally, the effect of PL on cell invasiveness was evaluated. MDA-MB-231 cells were plated on the Matrigel-coated filters in the presence of PL (0–1.0 mg ml^−1^) and the amount of cells invaded through Matrigel counted after 48 h of incubation. As seen in Figure 4C, PL inhibits invasion of MDA-MB-231 cells in a dose–response manner. Because cancer metastasis and invasiveness are associated with the uPA–uPA receptor (uPAR) system, we evaluated whether PL affects secretion of uPA from cancer cells. MDA-MB-231 cells were treated with PL (0–1.0 mg ml^−1^) for 24 h and uPA secretion evaluated by western blot analysis in conditioned media. In agreement with the data demonstrating inhibition of cell adhesion, migration and invasion PL markedly decreased secretion of uPA from MDA-MB-231 cells (Figure 4D).

### *Phellinus linteus* inhibits capillary morphogenesis of endothelial cells through the suppression of VEGF secretion

**Figure 5:**
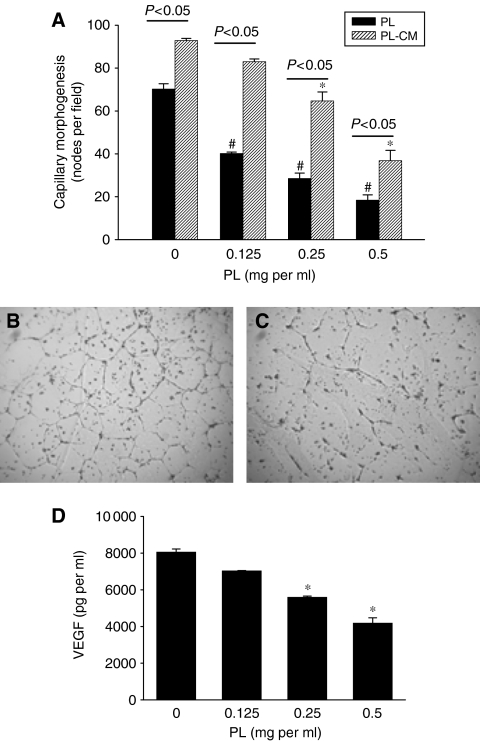
*Phellinus linteus* inhibits capillary morphogenesis of aortic endothelial cells. (**A**) HAECs were seeded onto Matrigel, and the cells were treated with PL (0–0.5 mg ml^−1^) (black bars) or with the conditioned media from MDA-MB-231 cells treated with PL (0–0.5 mg ml^−1^, PL-CM) (shaded bars) for 24 h and capillary morphogenesis was determined as described in Materials and Methods. Capillary morphogenesis at 0 mg ml^−1^ PL-CM (**B**) and at 0.5 mg ml^−1^ PL-CM (**C**). The number of nodules was quantified by counting from the three fields per data point and each bar represents the mean±s.d. of three experiments. Statistical analysis: black line *P*<0.05 PL (black bars) *vs* PL-CM (shaded bars) at the same concentration of PL, ^#^*P*<0.05 PL (black bars) at the PL concentration (0–0.5 mg ml^−1^), ^*^P<0.05 PL-CM (shaded bars) at the PL concentration (0–0.5 mg ml^−1^). (**D**) MDA-MB-231 cells were treated with PL (0–0.5 mg ml^−1^) for 24 h, media collected and secretion of VEGF determined as described in Materials and Methods. Each bar represents the mean±s.d. (pg per ml of secreted VEGF) of minimum three experiments repeated twice. ^*^P<0.05.

Capillary morphogenesis (tube formation) of human endothelial cells is one of the first important steps in angiogenesis associated with the cancer progression and metastasis. Because cancer microenvironment contains a variety of cells, we also evaluated if the inhibition of capillary morphogenesis of endothelial cells can be mediated through breast cancer cells. Therefore, we determined if PL itself or conditioned media from breast cancer cells exposed to PL suppress capillary morphogenesis of HAECs. Human aortic endothelial cells grown on Matrigel were treated directly with PL (0–0.5 mg ml^−1^) or with the conditioned media from MDA-MB-231 cells treated with PL (0–0.5 mg ml^−1^, PL-CM) for 24 h, and tube formation were evaluated. As seen in Figure 5A, PL significantly suppressed capillary morphogenesis of HAECs. Moreover, conditioned media from MDA-MB-231 cells without PL induced capillary morphogenesis of HAECs (PL *vs* PL-CM at 0 PL), and conditioned media from cells exposed to PL (PL-CM) also suppressed capillary morphogenesis of HAECs in a dose–response manner (Figure 5A–C). Because MDA-MB-231 cells express pro-angiogenic VEGF (Basu *et al*, 2005), we hypothesised that secreted VEGF from these cells induces capillary morphogenesis of endothelial cells, which can be inhibited by PL. Therefore, we evaluated whether PL inhibits secretion of VEGF from MDA-MB-231 cells treated with PL (0–0.5 mg ml^−1^). As seen in Figure 5D, secretion of VEGF from MDA-MB-231 cells was markedly decreased by PL in a dose–response manner. Collectively, our data suggest that PL inhibits capillary morphogenesis of endothelial cells directly as well as indirectly through the suppression of secretion of VEGF from breast cancer cells.

### *Phellinus linteus* inhibits activity of AKT kinase

**Figure 6:**
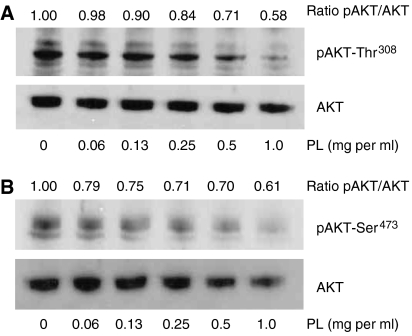
Effect of PL on AKT activity. MDA-MB-231 cells were treated with PL (0–1.0 mg ml^−1^) for 24 h and whole-cell extracts were subjected to western blot analysis with (**A**) anti-p-AKT-Thr^308^ or (**B**) anti-p-AKT-Ser^473^ antibodies. The equal protein loading was verified with anti-AKT antibody. The level of pAKT (ratio pAKT/AKT) was quantified by densitometry as described in Materials and Methods. The results are representative of three separate experiments.

AKT serine-threonine kinase (protein kinase B) regulates a variety of cellular processes through the phosphorylation of a wide spectrum of downstream substrates finally resulting in the expression of proteins involved in cell proliferation, invasiveness and angiogenesis among others (Woodgett, 2005; Dillon *et al*, 2007). To determine if PL modulates AKT activity in breast cancer cells, MDA-MB-231 cells were treated with PL (0–1.0 mg ml^−1^) for 24 h and the phosphorylation status of AKT evaluated in whole-cell extracts by western blot analysis. As seen in Figure 6A, PL inhibits phosphorylation of AKT at Thr^308^ in a dose–response manner. In addition, PL treatment also markedly decreased phosphorylation of AKT at Ser^473^ (Figure 6B).

**Figure 7:**
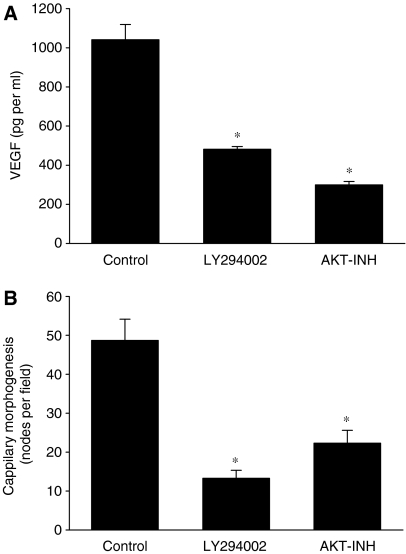
AKT inhibition suppresses VEGF secretion and capillary morphogenesis. MDA-MB-231 cells were treated with vehicle, LY294002 (10 *μ*M) or AKT inhibitor III (10 *μ*M) for 24 h and conditioned media collected. (**A**) Vascular endothelial growth factor secretion from MDA-MB-231 cells and (**B**) capillary morphogenesis of HEAC was determined as described in Materials and Methods. Each bar represents the mean±s.d. of minimum three experiments repeated twice. ^*^P<0.05.

To evaluate if the suppression of AKT activity PL is directly responsible for the inhibition of capillary morphogenesis through the downregulation of expression of VEGF in MDA-MB-231 cells, we used a pharmacological approach with specific AKT inhibitors. Therefore, MDA-MB-231 cells were treated with LY294002 (10 *μ*M) or AKT inhibitor III (10 *μ*M) for 24 h, and secretion of VEGF was evaluated. As seen in Figure 7A, AKT inhibitors markedly suppressed secretion of VEGF from breast cancer cells. Moreover, conditioned media from MDA-MB-231 cells treated with LY294002 and AKT inhibitor III also inhibited capillary morphogenesis of endothelial cells (Figure 7B). Therefore, these data suggest that the inhibition of angiogenesis *in vitro* by PL is mediated, to some extent, through the suppression of AKT activity, which results in the suppression of secretion of VEGF from breast cancer cells.

## DISCUSSION

Regardless of different theories of biology of breast cancer as (i) a local disease that spreads over time to develop distant metastases; (ii) a systemic disease from the outset, with distant metastases present well before diagnosis (iii) or the combination of both as a heterogeneous disease, cancer metastasis is one of the major medical problems in breast cancer patients (Punglia *et al*, 2007). While several chemotherapeutic agents or their combinations (e.g. taxanes, trastumazab, gemcitabine or capecitabine) demonstrated activity in the metastatic breast cancer setting (Tripathy, 2007), there is a paucity of natural antiproliferative and anti-metastatic nontoxic agents.

As demonstrated practically four decades ago, polysaccharide extracts from basidiomycete fungus PL suppressed tumour growth *in vivo* (Chihara *et al*, 1969). In addition, PL also reduced tumour growth and the frequency of pulmonary metastasis without toxic effects (Han *et al*, 1999). Recent studies elucidated some of the molecular mechanism(s) responsible for the inhibition of growth through cell cycle arrest and induction of apoptosis in lung and prostate cancer cells (Collins *et al*, 2006; Guo *et al*, 2007; Zhu *et al*, 2007). Nevertheless, the molecular mechanism(s) responsible for the inhibition of invasive behaviour and angiogenesis was not fully addressed.

In the present study, we demonstrate that PL inhibits cell proliferation (anchorage-dependent growth) as well as colony formation (anchorage-independent growth) of highly invasive breast cancer cells through the S-phase cell cycle arrest mediated by the upregulation of expression of p27. Cell growth, which is the reflection of the progression of cell cycle, is aberrantly regulated in the majority of cancers. The cell cycle is regulated by a series of checkpoints employing cyclins, cdks and cdk inhibitors (Sherr, 1966). p27 is one of the cdk inhibitors, which binds to S-phase cyclin–cdk complexes and inhibits their cell cycle stimulatory activities. Because a loss of p27 expression has been linked to the aggressive behaviour in a variety of human epithelial tumours including breast cancer, the induction of p27 expression could lead to cell cycle arrest and inhibition of tumour growth (Tan *et al*, 1997; Lloyd *et al*, 1999; Macri and Loda, 1999). Our data are in agreement with recent papers also demonstrating the inhibition of growth of prostate cancer and leukaemia cells through the S-phase cell cycle arrest by the upregulation of expression of p27 (Zhang *et al*, 2006; Shishodia *et al*, 2007). Recently, Guo *et al* (2007) demonstrated that PL suppresses growth of lung cancer cells through G1-phase cell cycle arrest mediated by the inhibition of cdk2, 4 and 6 activities. Our results show cell cycle arrest at S phase in breast cancer cells through the upregulation of p27. Nevertheless, our data are not in a disagreement with Guo *et al* because the passage through G1 phase into S phase is regulated by the activities of cdk2, 4 and 6, which are controlled by cdk inhibitor p27 (Slingerlan and Pagano, 2000). Moreover, cell cycle arrest at S phase can also be interpreted as the arrest at G1-S, because the majority of cells are at G0/G1 and S but not G2 phases (Table 1). Most importantly, PL suppresses growth of cancer cells by the cell cycle arrest.

Here, we show that PL inhibits adhesion, migration and invasion through the suppression of secretion of uPA from highly invasive breast cancer cells. Our data are in agreement with the study by Lee *et al* (2005) demonstrating the inhibition of adhesion, invasion and expression of uPA in mouse melanoma cells. Furthermore, our data suggest the mechanism of inhibition of invasiveness by PL. Therefore, secreted uPA from breast cancer cells interacts with uPAR and converts plasminogen to plasmin (Blasi and Carmeliet, 2002). Plasmin degrades ECM components and stimulates other proteolytic enzymes (MMPs), which through the degradation of ECM contribute to cell invasion (Blasi and Carmeliet, 2002). Secreted uPA can bind to uPAR and forms a complex with integrin receptor *α*_V_*β*_3_, which through its interaction with vitronectin is involved in adhesion and migration of breast cancer cells (Slivova *et al*, 2005). Inhibition of uPA secretion will reduce the formation of uPA–uPAR–*α*_V_*β*_3_–vitronectin complex, with the consequent suppression of adhesion and migration of invasive breast cancer cells. Alternatively, PL can also modulate activities of other proteins involved in the invasive behaviour of breast cancer cells (e.g. matrix metalloproteinases, *β*_1_ and *β*_4_ integrins, epidermal growth factor receptors and others (Carraway and Sweeney, 2006; Bissell, 2007)). Nevertheless, in the present study, we propose that PL suppresses invasiveness through the inhibition of uPA secretion. Finally, we and others have previously demonstrated that inhibition of uPA suppressed invasiveness of breast cancer cells (Sliva *et al*, 2002b; Das *et al*, 2003: Mi *et al*, 2006).

Recently, Song *et al* (2003) demonstrated anti-angiogenic activity of PL in chorioallantoic membrane (CAM) chick embryo assay. However, the mechanism of the inhibition of angiogenesis by PL, related to cancer, was not previously addressed. In the present study, we demonstrate that PL inhibits one of the first steps in angiogenesis – tube formation of endothelial cells. While PL directly suppressed capillary morphogenesis of endothelial cells, our data further suggest that this effect can be mediated by the inhibition of secretion of VEGF from breast cancer cells. Although in our experimental conditions it was impossible to remove PL from the conditioned media from breast cancer cells (PL-CM), and therefore their inhibitory effect on capillary morphogenesis of HEACs could be considered as a direct effect of PL on HEACs, our data suggest that both (direct and indirect) effects are involved. Thus, (i) conditioned media from breast cancer cells without PL significantly increased capillary morphogenesis of endothelial cells, suggesting that proangiogenic factor is released from breast cancer cells, (ii) PL itself as well as conditioned media containing PL suppressed capillary morphogenesis and (iii) the secretion of VEGF from breast cancer cells was inhibited by PL. In agreement with our observation, suppression of endothelial capillary morphogenesis through the inhibition of secreted VEGF from a variety of cancer cells was described recently (Fukumoto *et al*, 2005; Stanley *et al*, 2005; Jang *et al*, 2007; Kong *et al*, 2007). Therefore, inhibition of specific pro-angiogenic protein within cancer cells will affect the whole cancer microenvironment (containing different cells) and will finally result in the suppression of tumour angiogenesis.

One of the suitable molecular cancer targets is AKT kinase, which inhibition in breast cancer cells resulted in cell cycle arrest, inhibition of growth and colony formation, inhibition of migration, invasion and suppression of angiogenesis (Das *et al*, 2003; Yacoub *et al*, 2003; Jiang *et al*, 2004a; Basu *et al*, 2005; Fukumoto *et al*, 2005; Jallal *et al*, 2007). Our data clearly demonstrate that PL suppresses AKT activity through the inhibition of AKT phosphorylation at Thr^308^ and at Ser^473^ in MDA-MB-231 cells, which demonstrate high levels of constitutively active AKT. Furthermore, inhibition of AKT with LY294002 and more specific AKT inhibitor III suppressed secretion of VEGF from breast cancer cells resulting in the decrease of capillary morphogenesis of endothelial cells. Our observation is in agreement with Xia *et al* (2006) who demonstrated, by using siRNA against AKT, the downregulation of VEGF expression in ovarian cancer cells, and the inhibition of angiogenesis in CAM chick embryo assay.

In conclusion, our study suggests PL as a natural compound possessing antiproliferative, antimetastatic and anti-angiogenic effects, which could be considered for the therapy of invasive breast cancers. However, further studies are necessary to confirm and evaluate these anticancer effects *in vivo*.

## Figures and Tables

**Table 1:** Effect of PL on cell cycle distribution

**Time (h)**	**PL (mg per ml)**	**G0/G1**	**S**	**G2/M**	**subG0/G1**
0	0	48±0.6	26±0.4	26±0.4	0.5±0.04
24	0	52±0.5	30±0.7	17±0.2	2.3±0.10
48	0	51±1.1	30±0.3	19±0.9	0.7±0.09
0	0.5	47±2.7	27±1.2	26±1.9	1.2±0.36
24	0.5	47±1.3	34±1.1^*^	18±0.2	0.9±0.37
48	0.5	39±1.7	44±1.7^*^	17±0.9	1.1±0.20

Cell cycle distribution G0/G1, S, G2/M and subG0/G1 in %.

^*^Statistical significance *P*<0.005 for cells at S phase (24 and 48 h) *vs* control (0 h) from three experiments.
